# Supplementary figures and images for: Centromeres in budding yeasts are conserved in chromosomal location but not in structure
Source: PLoS Genet. 2025 Dec 8;21(12):e1011814. doi: 10.1371/journal.pgen.1011814 (PMC12711049; doi:10.1371/journal.pgen.1011814)

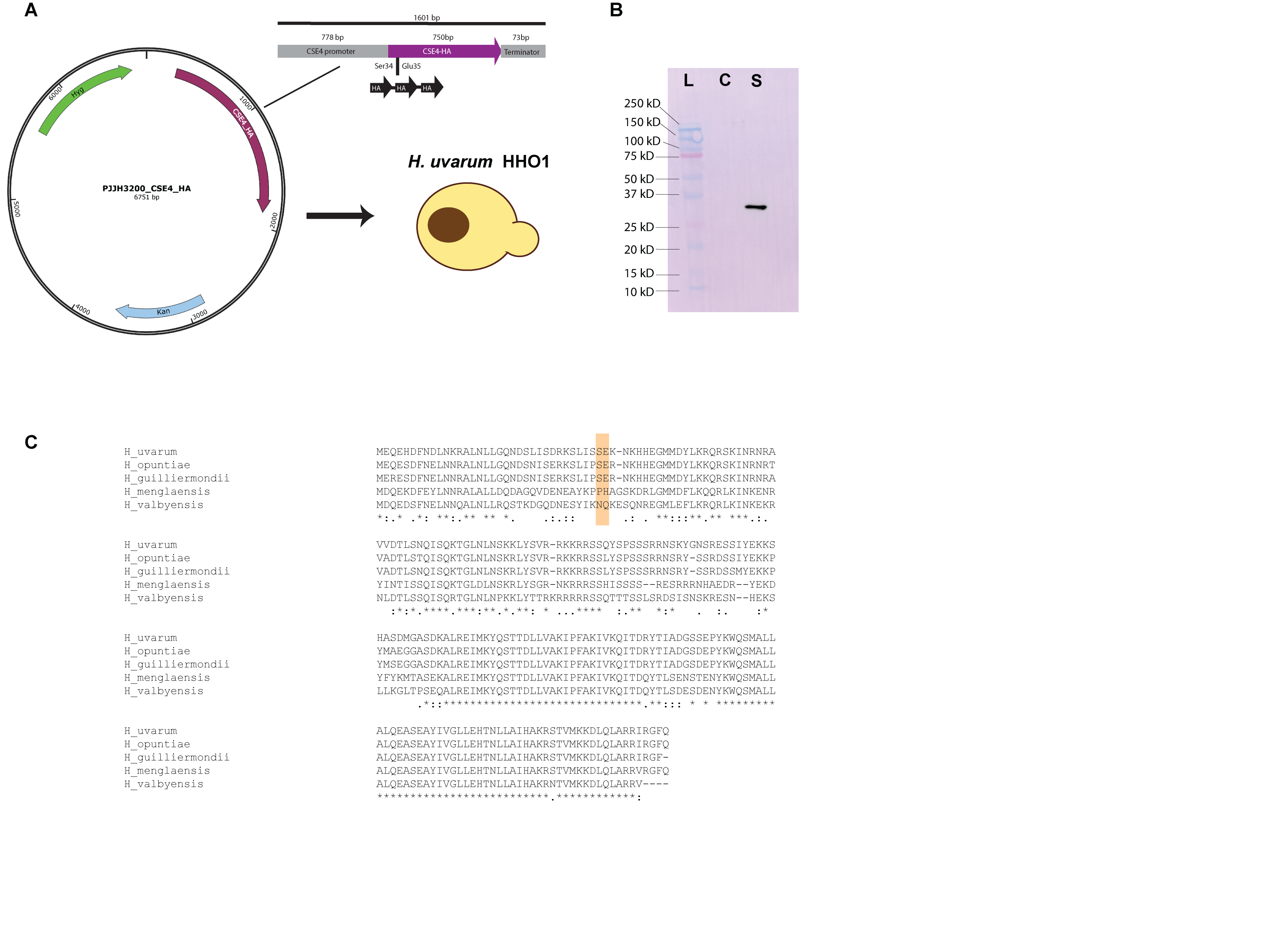

Supplement: S1 Fig — Construction of an H. uvarum strain expressing an HA-tagged Cse4 protein for ChIP-seq analysis. A. A synthetic gene coding for HuCse4 with 3 HA tags between codons 34 and 35 was cloned into plasmid pJJH3200 [42] and transformed into H. uvarum strain HHO1 by electroporation.B. Expression of HuCse4-HA in H. uvarum was detected by Western blot using anti-HA antibody. Lane L: Precision Plus Protein Dual Color Standards (Bio-RAD). Sizes are shown in kD. Lane C: Protein extract from untransformed H. uvarum HHO1. Lane S: Protein extract from transformed H. uvarum HHO1 strain expressing pJJH3200_CSE4_HA plasmid. C. Multiple sequence alignment of Cse4 proteins from five Hanseniaspora species, showing the site at which the 3xHA tag was inserted in H. uvarum Cse4. (TIF) [file pgen.1011814.s001.tif]

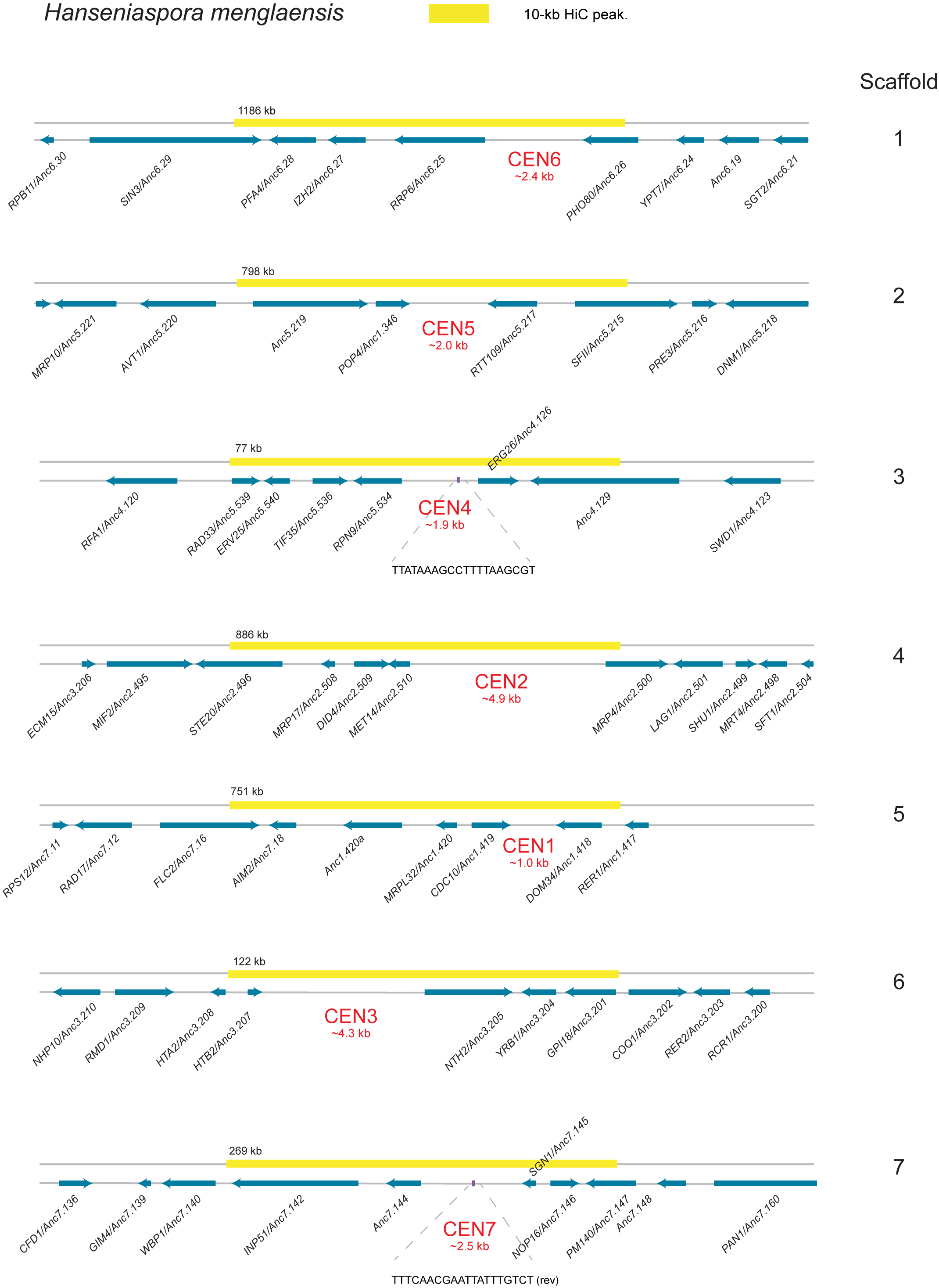

Supplement: S2 Fig — Yellow boxes show the locations of the 10-kb Hi-C peak windows identified on each scaffold, with the start position (kb) of each window labeled on its left. Blue arrows show annotated genes, named according to their S. cerevisiae orthologs and ancestral (Anc) gene numbers where possible. The location of motifs at CEN4 and CEN7 matching those found at H. uvarum centromeres are shown. The CEN numbers reflect the ancestral centromere numbering that has been applied to all species. The Scaffold numbers are specific to the H. menglaensis (Hmeng) assembly. (TIF) [file pgen.1011814.s002.tif]

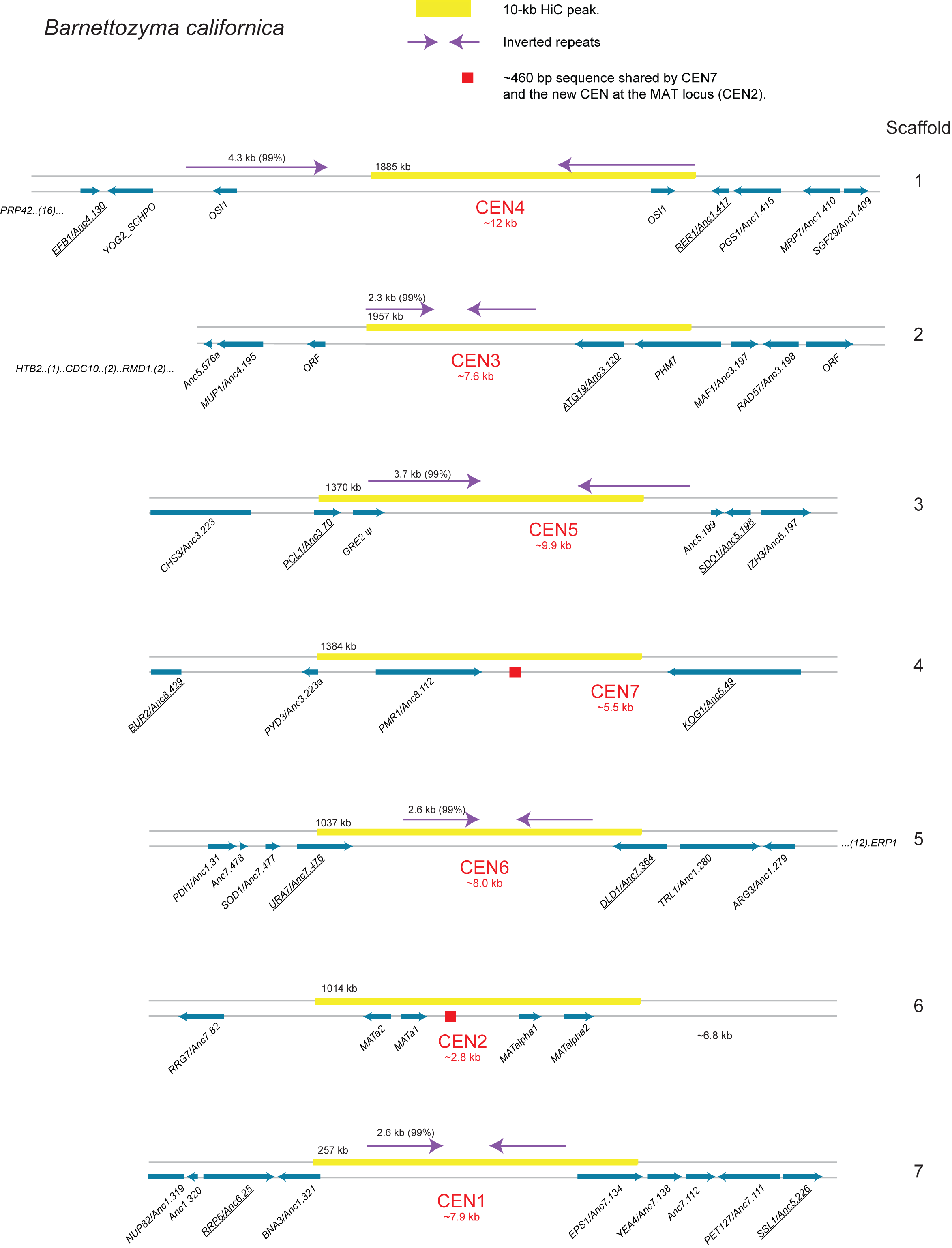

Supplement: S3 Fig — Yellow boxes show the locations of the 10-kb Hi-C peak windows identified on each scaffold, with the start position (kb) of each window labeled on its left. Magenta arrows show the location of large Inverted Repeats (IRs), with their length and percent sequence identity shown. The red boxes indicate a ~ 460 bp sequence shared by CEN7 (Scaffold 4) and CEN2 (Scaffold 6). Blue arrows show annotated genes, named according to their S. cerevisiae orthologs and ancestral (Anc) gene numbers where possible. The CEN numbers reflect the ancestral centromere numbering that has been applied to all species. The Scaffold numbers are specific to the B. californica (Barcal) assembly. Genes whose names are underlined were used as landmarks in Fig 6; other landmark genes that lie outside the illustrated region are named at the edges, with the number of ORFs separating them shown in parentheses. (TIF) [file pgen.1011814.s003.tif]

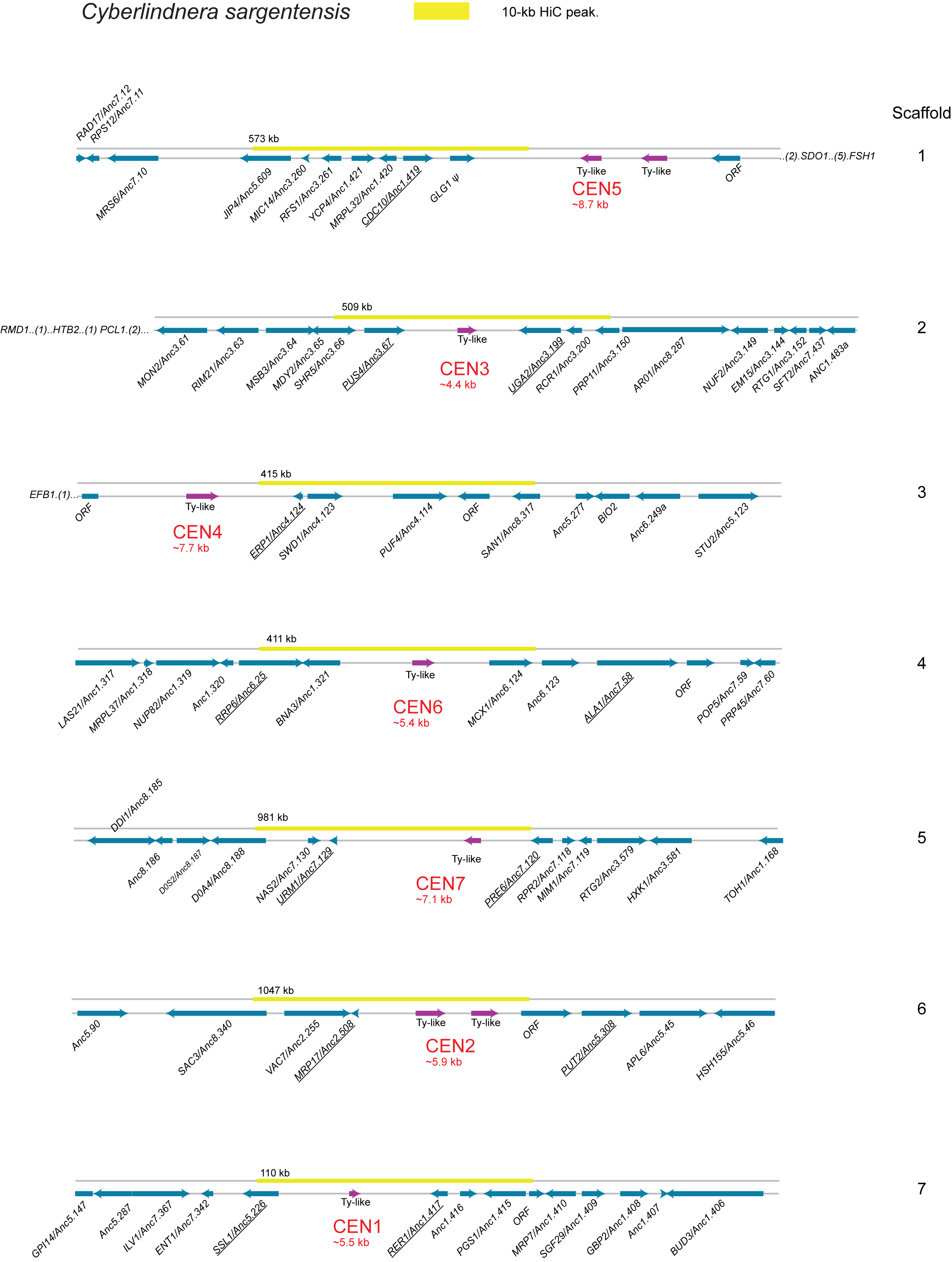

Supplement: S4 Fig — Yellow boxes show the locations of the 10-kb Hi-C peak windows identified on each scaffold, with the start position (kb) of each window labeled on its left. Magenta arrows show sequences annotated as Ty5-like pseudogenes. Blue arrows show annotated genes, named according to their S. cerevisiae orthologs and ancestral (Anc) gene numbers where possible. The CEN numbers reflect the ancestral centromere numbering that has been applied to all species. The Scaffold numbers are specific to the C. sargentensis (Cybsar) assembly. Genes whose names are underlined were used as landmarks in Fig 6; other landmark genes that lie outside the illustrated region are named at the edges, with the number of ORFs separating them shown in parentheses. (TIF) [file pgen.1011814.s004.tif]

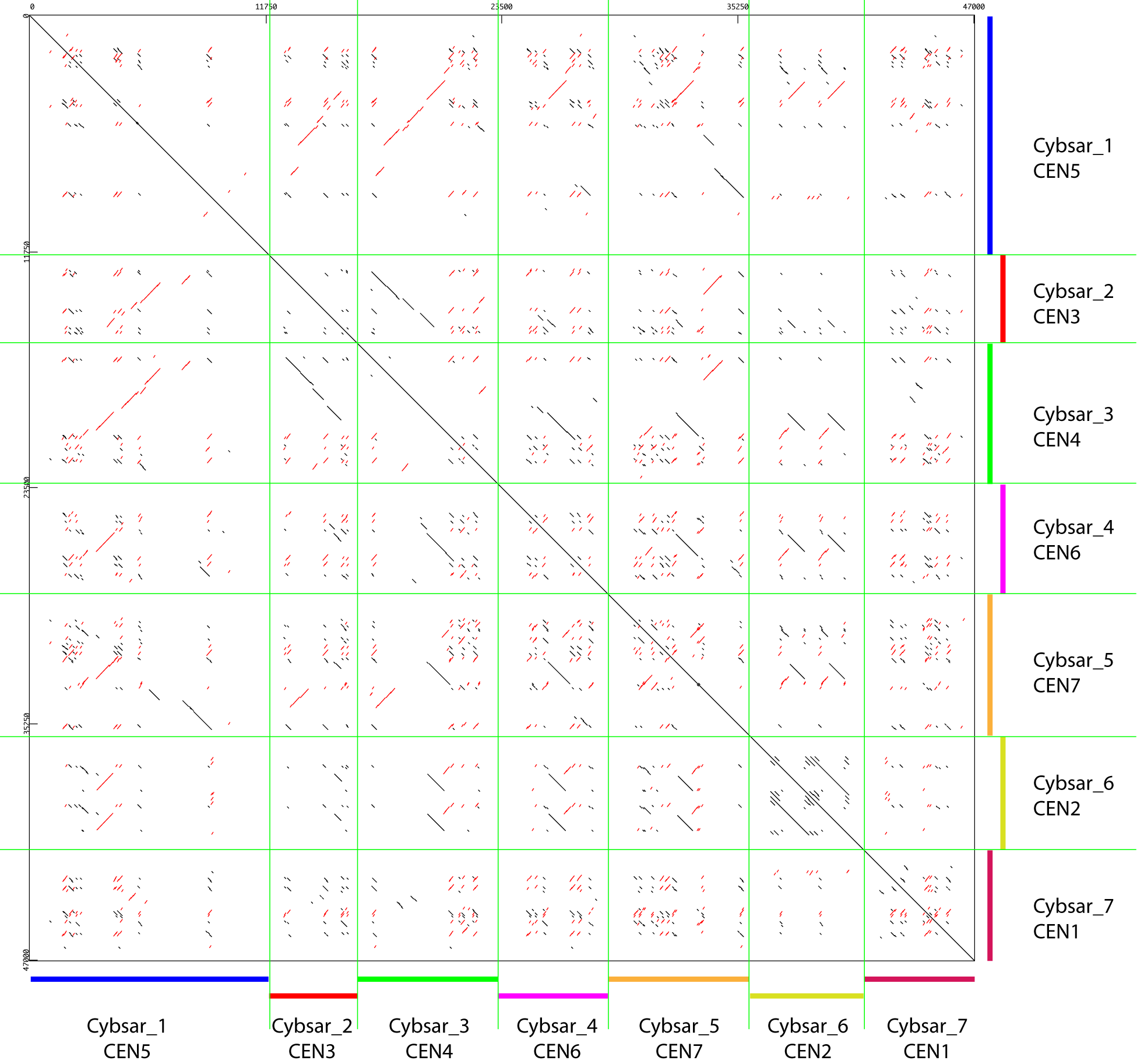

Supplement: S5 Fig — The sequences of the longest intergenic region within the Hi-C peaks on each chromosome were extracted and concatenated. The plot was generated using DNAMAN with a threshold of 17 mismatches per 56-bp window. (TIF) [file pgen.1011814.s005.tif]

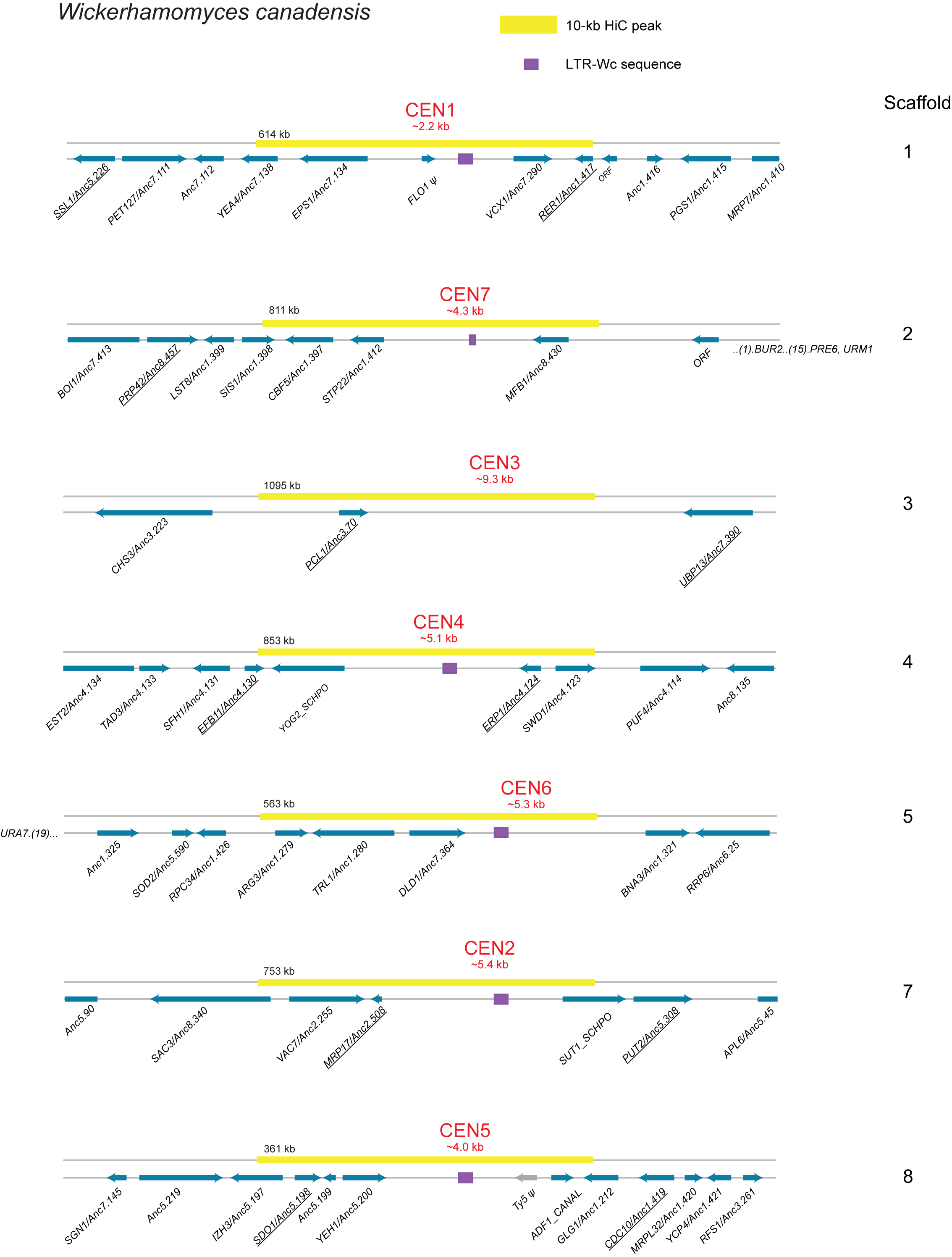

Supplement: S6 Fig — Yellow boxes show the locations of the 10-kb Hi-C peak windows identified on each scaffold, with the start position (kb) of each window labeled on its left. Magenta boxes show sequences derived from LTR-Wc (see Fig 5B for detail), which are present at every centromere except CEN3. Blue arrows show annotated genes, named according to their S. cerevisiae orthologs and ancestral (Anc) gene numbers where possible. The CEN numbers reflect the ancestral centromere numbering that has been applied to all species. The Scaffold numbers are specific to the W. canadensis (Wcanad) assembly. Genes whose names are underlined were used as landmarks in Fig 6; other landmark genes that lie outside the illustrated region are named at the edges, with the number of ORFs separating them shown in parentheses. (TIF) [file pgen.1011814.s006.tif]

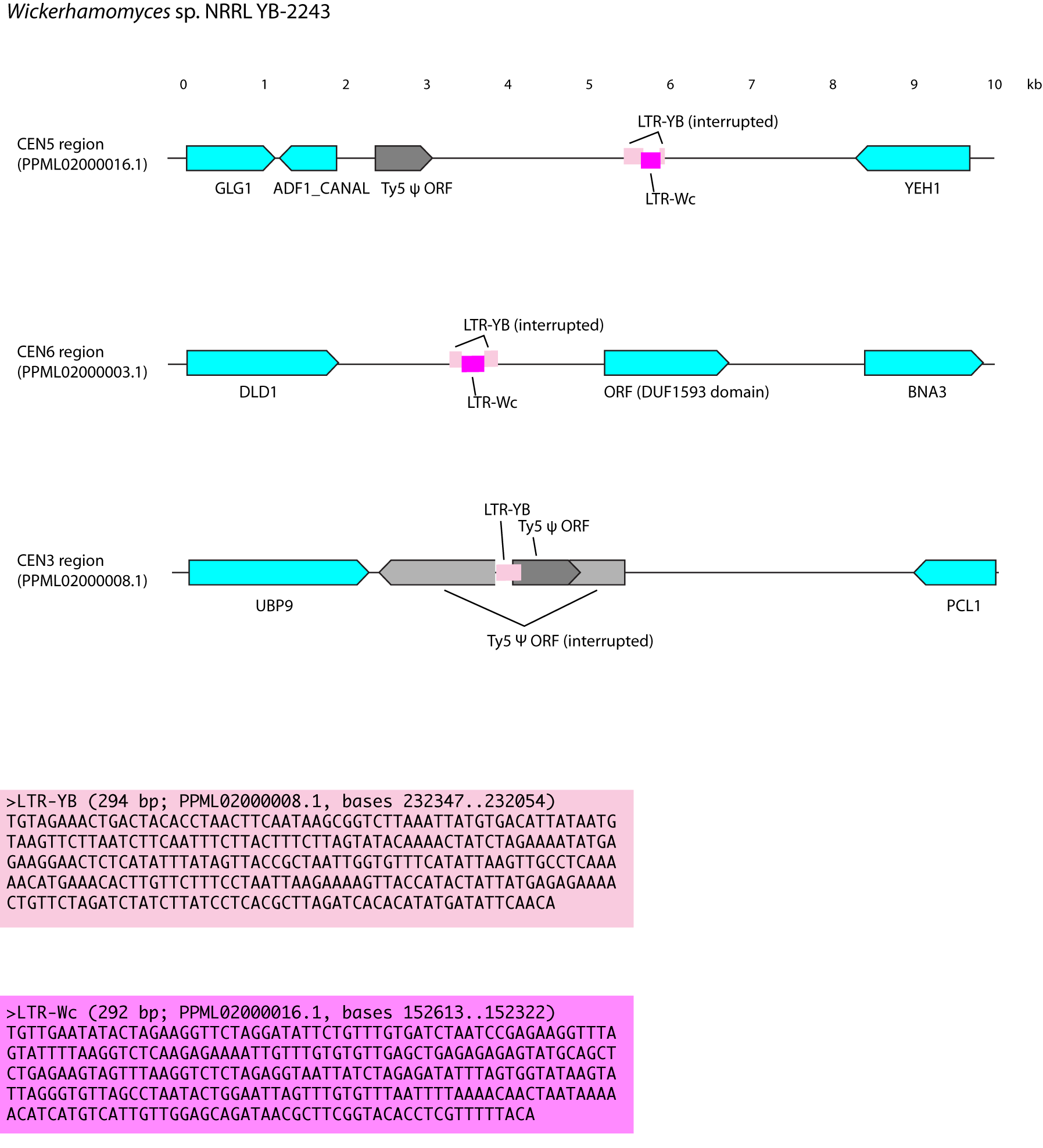

Supplement: S7 Fig — Examples of LTR-containing regions from CEN5, CEN6 and CEN3 are shown. At CEN5 and CEN6, copies of LTR-Wc have become inserted into LTR-YB. LTR-Wc has sequence similarity to the 200-bp motif identified in centromeres of W. canadensis. LTR-YB is present in Wickerhamomyces sp. NRRL YB-2243 but not in W. canadensis CBS 1992. (TIF) [file pgen.1011814.s007.tif]

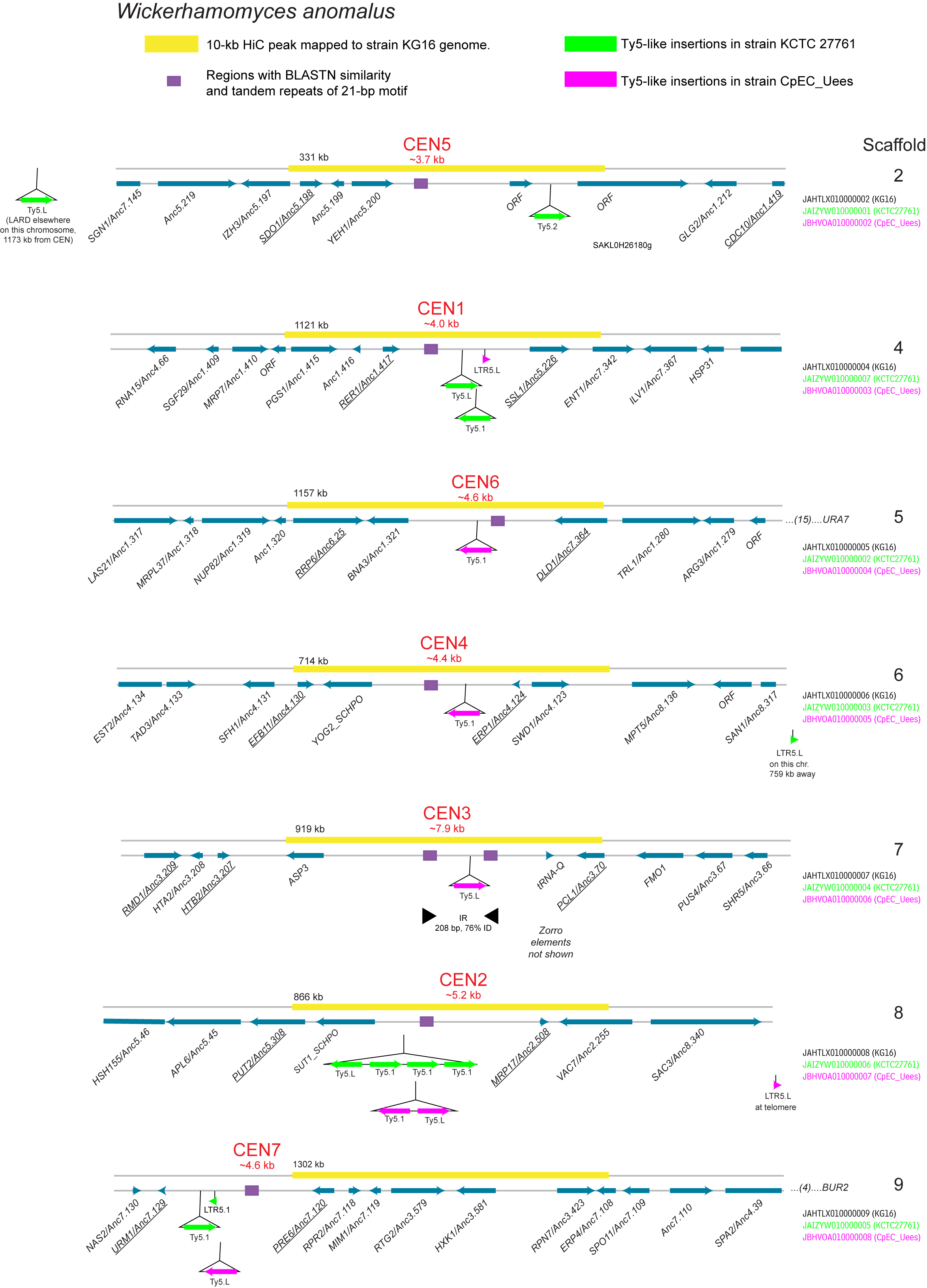

Supplement: S8 Fig — Yellow boxes show the locations of the 10-kb Hi-C peak windows identified on each scaffold, with the start position (kb) of each window labeled on its left. Magenta boxes show sequence regions that were initially identified by BLASTN as being similar among centromeres, and which contain multiple iterations of a 21-bp motif (Fig 5C). The gene maps and coordinates are from the assembly of the primary haplotype of W. anomalus isolate KG16, which does not contain any identified Ty5-like elements or LTRs. Two other isolates of W. anomalus, KCTC 27761 (green) and CpEC_Uees (magenta) contain three types of Ty5-like element (labeled Ty5.1, Ty5.2 and Ty5.L) integrated at different sites as shown. NCBI accession numbers are shown for each isolate. Blue arrows show annotated genes, named according to their S. cerevisiae orthologs and ancestral (Anc) gene numbers where possible. The CEN numbers reflect the ancestral centromere numbering that has been applied to all species. The Scaffold numbers are specific to the W. anomalus (Wanom) KG16 assembly. Genes whose names are underlined were used as landmarks in Fig 6; other landmark genes that lie outside the illustrated region are named at the edges, with the number of ORFs separating them shown in parentheses. (TIF) [file pgen.1011814.s008.tif]

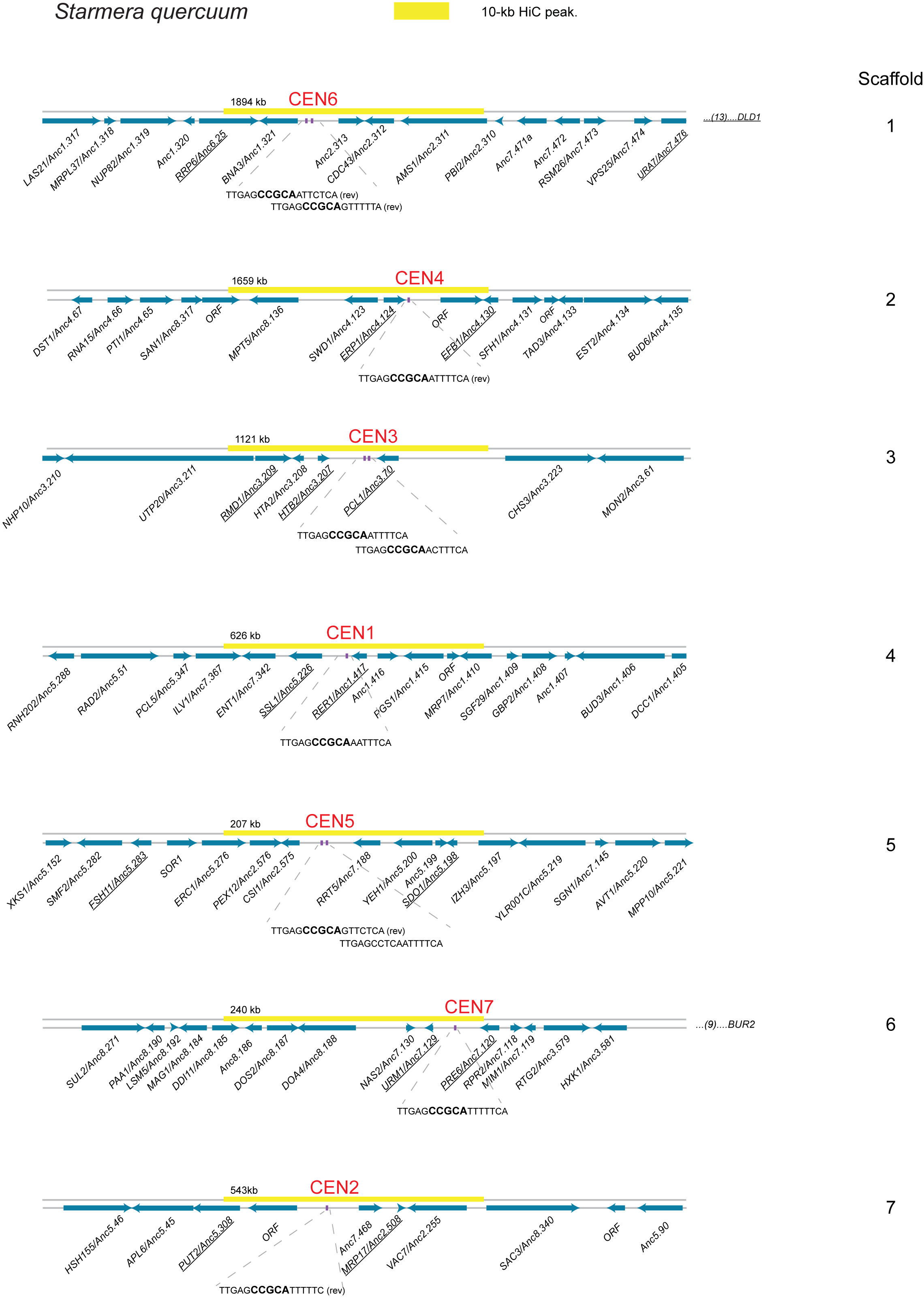

Supplement: S9 Fig — Yellow boxes show the locations of the 10-kb Hi-C peak windows identified on each scaffold, with the start position (kb) of each window labeled on its left. Magenta boxes show occurrences of the 17-bp motif identified in Fig 5D. Blue arrows show annotated genes, named according to their S. cerevisiae orthologs and ancestral (Anc) gene numbers where possible. The CEN numbers reflect the ancestral centromere numbering that has been applied to all species. The Scaffold numbers are specific to the S. quercuum (Staque) assembly. Genes whose names are underlined were used as landmarks in Fig 6; other landmark genes that lie outside the illustrated region are named at the edges, with the number of ORFs separating them shown in parentheses. (TIF) [file pgen.1011814.s009.tif]

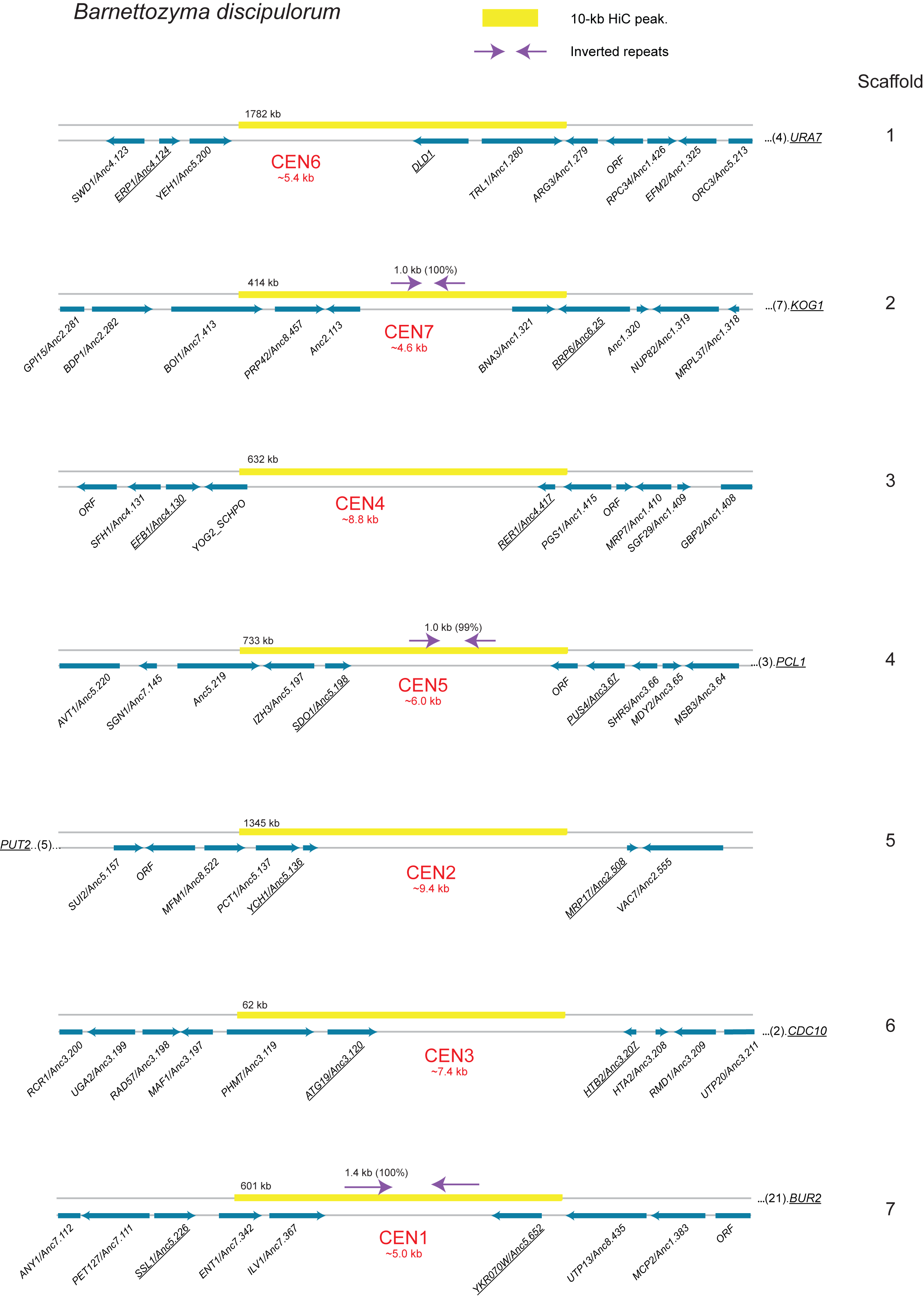

Supplement: S10 Fig — Yellow boxes show the locations of the 10-kb Hi-C peak windows identified on each scaffold, with the start position (kb) of each window labeled on its left. Magenta arrows show the location of large Inverted Repeats (IRs), with their length and percent sequence identity shown. Blue arrows show annotated genes, named according to their S. cerevisiae orthologs and ancestral (Anc) gene numbers where possible. The CEN numbers reflect the ancestral centromere numbering that has been applied to all species. The Scaffold numbers are specific to the B. discipulorum (Bardis) assembly. Genes whose names are underlined were used as landmarks in Fig 6; other landmark genes that lie outside the illustrated region are named at the edges, with the number of ORFs separating them shown in parentheses. (TIF) [file pgen.1011814.s010.tif]

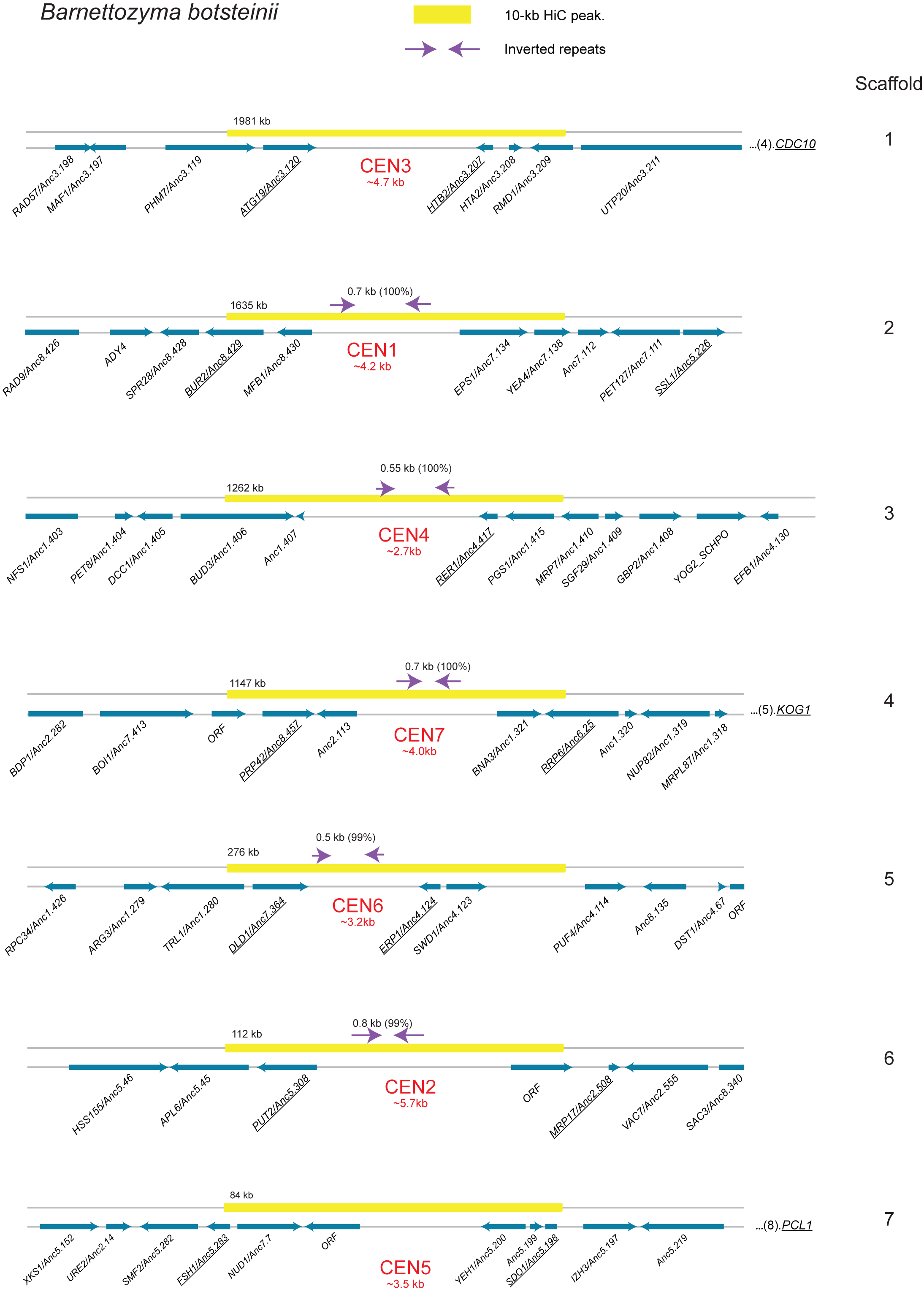

Supplement: S11 Fig — Yellow boxes show the locations of the 10-kb Hi-C peak windows identified on each scaffold, with the start position (kb) of each window labeled on its left. Magenta arrows show the location of large Inverted Repeats (IRs), with their length and percent sequence identity shown. Blue arrows show annotated genes, named according to their S. cerevisiae orthologs and ancestral (Anc) gene numbers where possible. The CEN numbers reflect the ancestral centromere numbering that has been applied to all species. The Scaffold numbers are specific to the B. botsteinii (Barbot) assembly. Genes whose names are underlined were used as landmarks in Fig 6; other landmark genes that lie outside the illustrated region are named at the edges, with the number of ORFs separating them shown in parentheses. (TIF) [file pgen.1011814.s011.tif]

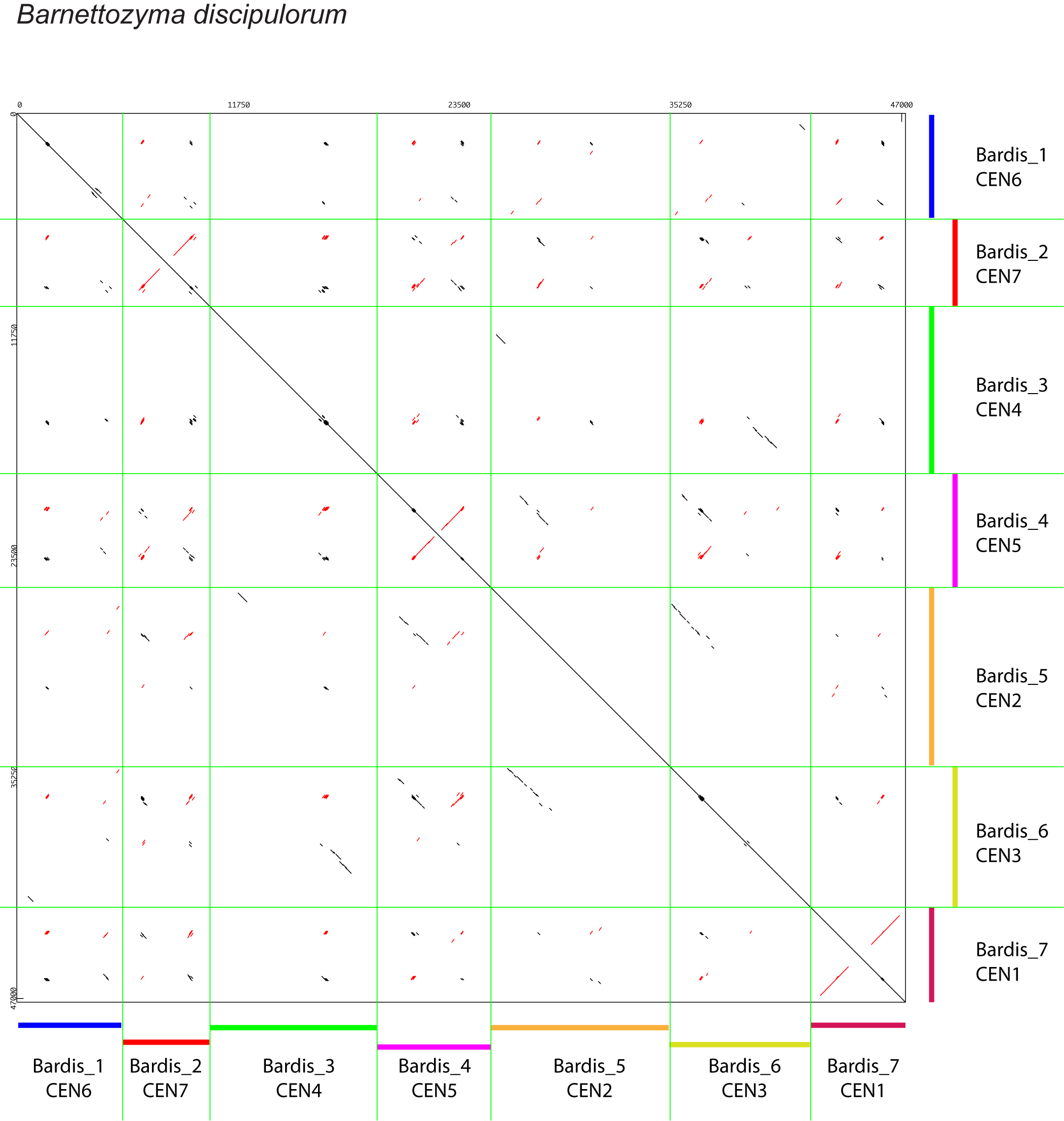

Supplement: S12 Fig — The sequences of the longest intergenic region within the Hi-C peaks on each chromosome were extracted and concatenated. The plot was generated using DNAMAN with a threshold of 17 mismatches per 56-bp window. (TIF) [file pgen.1011814.s012.tif]

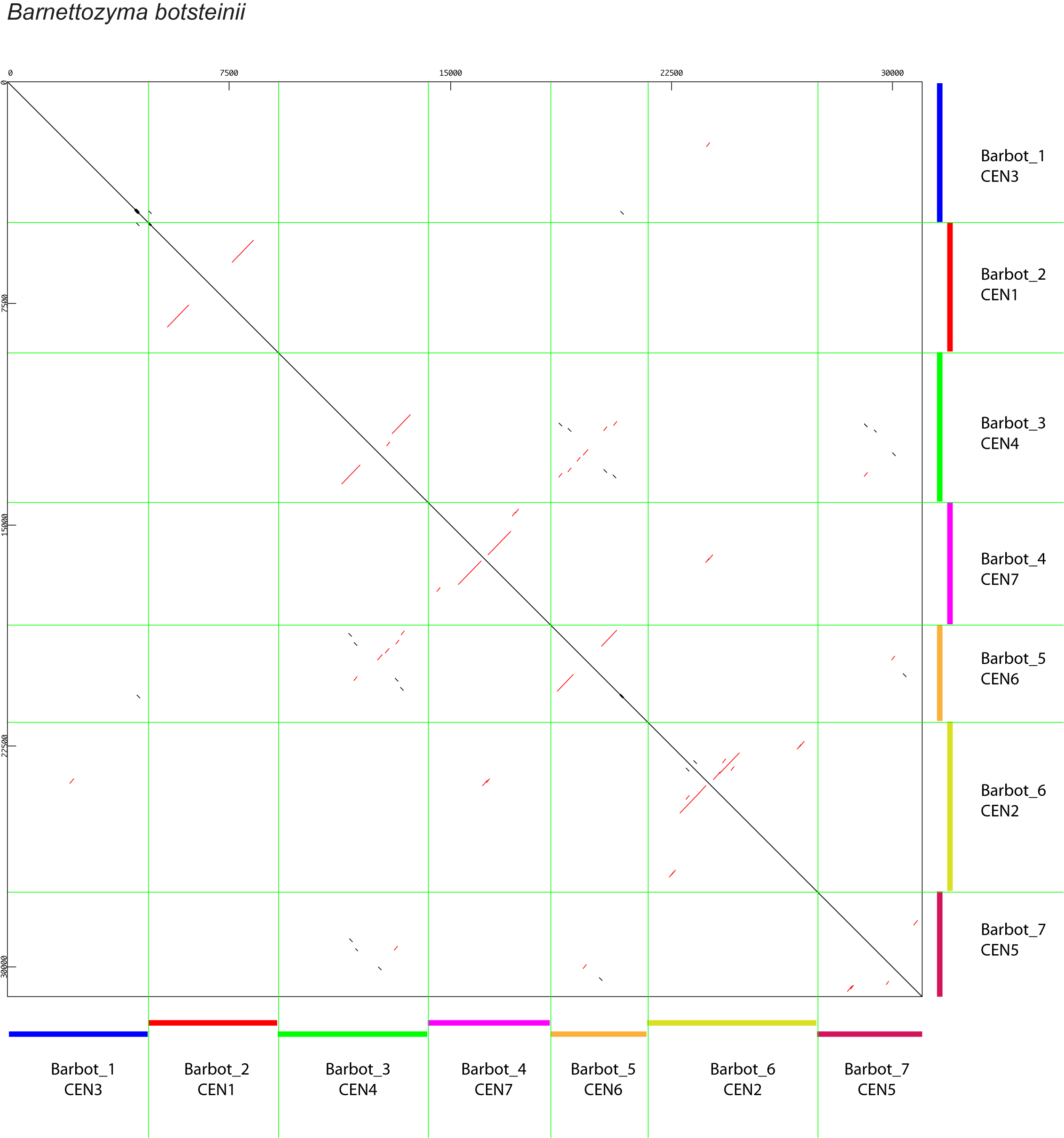

Supplement: S13 Fig — The sequences of the longest intergenic region within the Hi-C peaks on each chromosome were extracted and concatenated. The plot was generated using DNAMAN with a threshold of 17 mismatches per 56-bp window. (TIF) [file pgen.1011814.s013.tif]

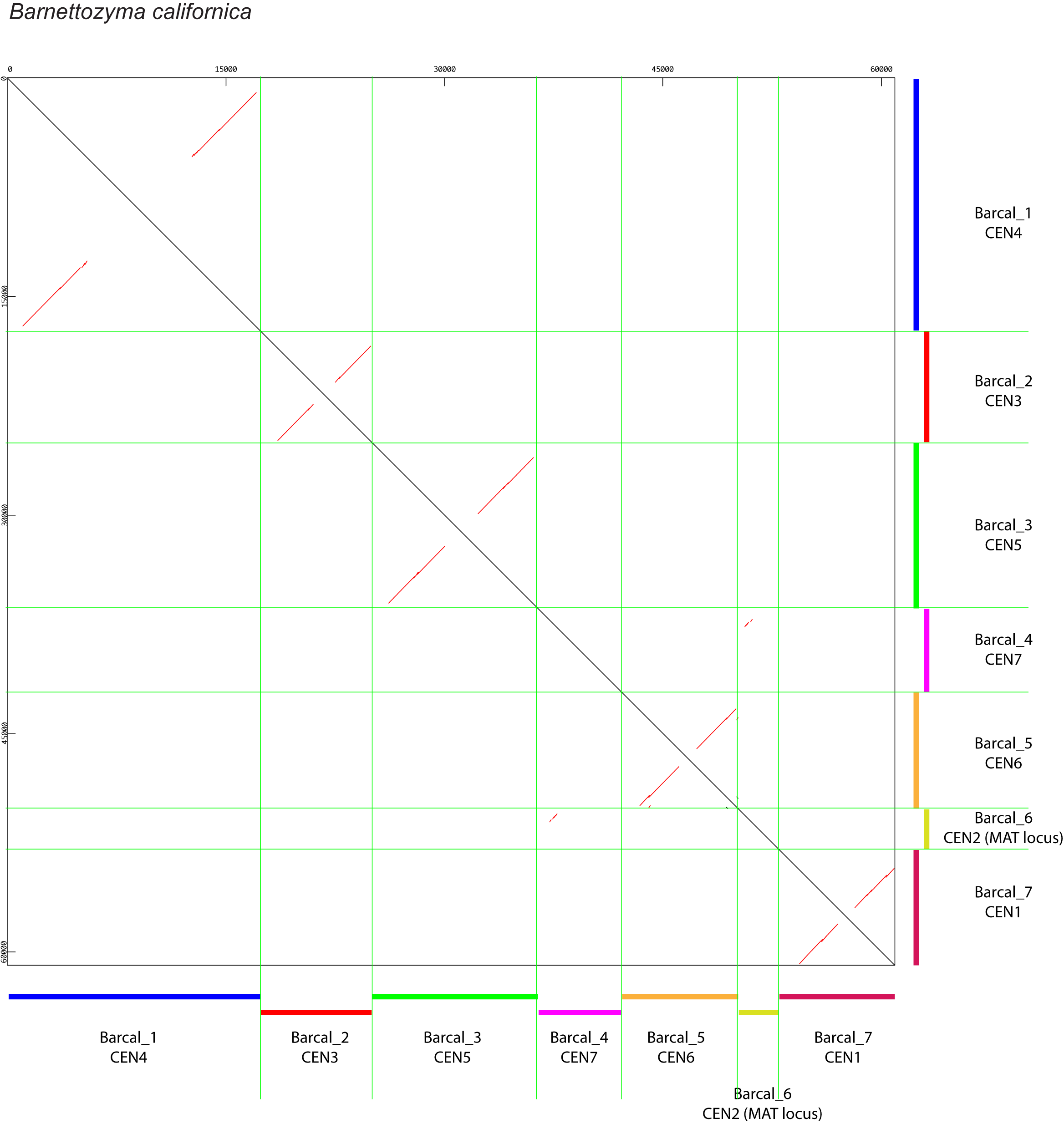

Supplement: S14 Fig — The sequences of the longest intergenic region within the Hi-C peaks on each chromosome were extracted and concatenated. The plot was generated using DNAMAN with a threshold of 17 mismatches per 56-bp window. (TIF) [file pgen.1011814.s014.tif]

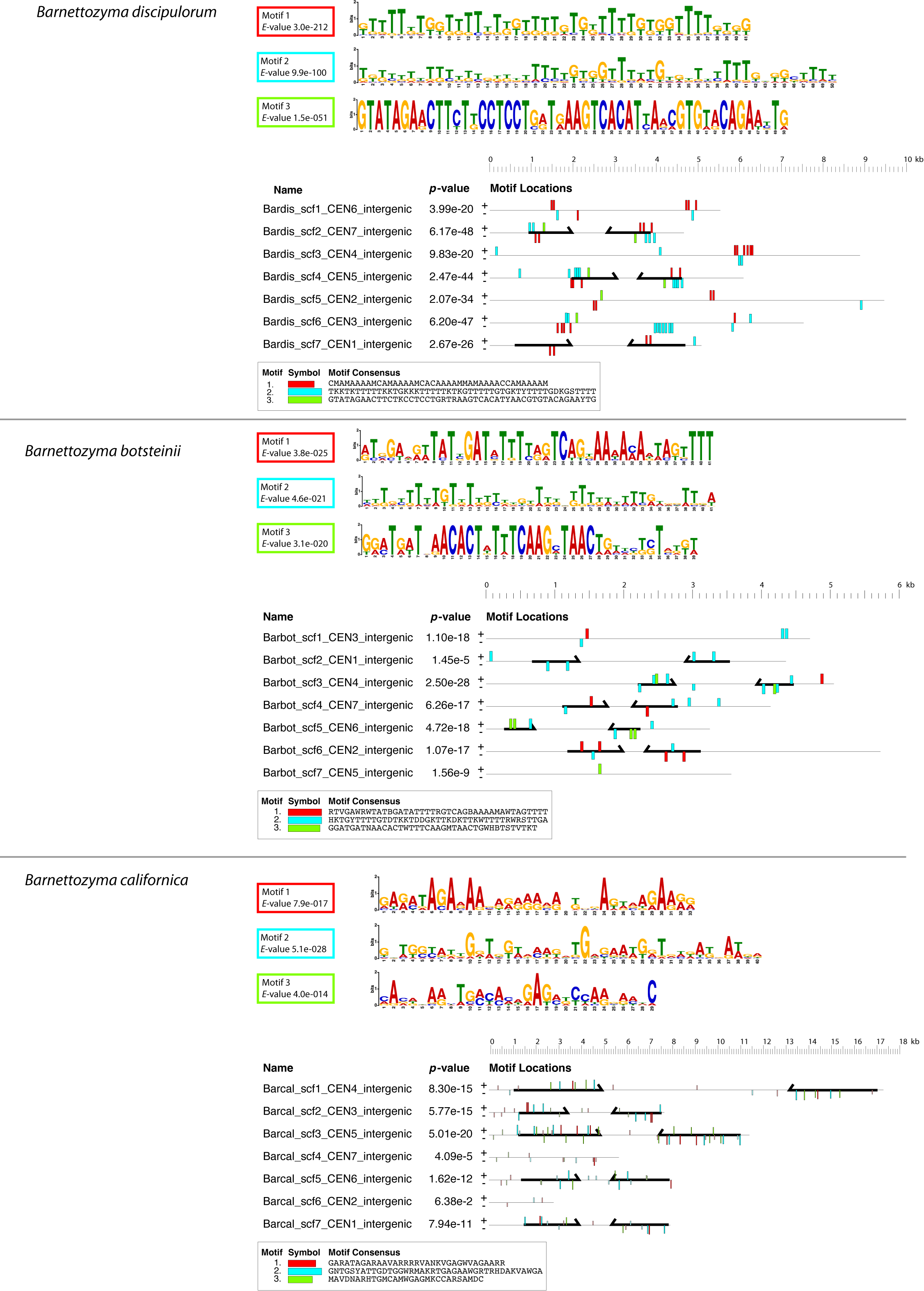

Supplement: S15 Fig — For each species, the input sequences were the largest intergenic region at each Hi-C peak. MEME was run using default parameters and the ANR (“any number of repeats”) option, and identified the three most significantly enriched motifs. The locations of the motifs, relative to the Inverted Repeats (black arrows) present at some centromeres, are shown. In B. discipulorum, Motifs 1 consists of tandem iterations of an 8-mer with the consensus sequence GTGGTTTT, and occurs on all chromosomes. Motif 2 is a variant of Motif 1. (TIF) [file pgen.1011814.s015.tif]
