# Supplementary material for: Centromeres in budding yeasts are conserved in chromosomal location but not in structure
Source: PLoS Genet. 2025 Dec 8;21(12):e1011814. doi: 10.1371/journal.pgen.1011814 (PMC12711049; doi:10.1371/journal.pgen.1011814)
Supplement: S1 Table — (PDF) [file pgen.1011814.s016.pdf]

Table S1. Sources of genome sequence data, Hi-C data, and ChIP-seq data used in this study.

**Species**	**Genome**					**Hi-C data**			**Cse4 ChIP-seq data**		
	Isolate used for genome analysis and Hi-C mapping	Number of scaffolds (excl. mtDNA)	Assembly size (Mb)	Accession number for genome assembly	Reference for genome sequence	Reference for Hi-C data	Isolate used for HI-C sequencing	SRA accesion number for Hi-C data	Reference for ChIP-seq	Isolate used for ChIP-seq	SRA accession numbers for ChIP-seq
Hanseniaspora uvarum	CBA6001	8	9.0	PTQS01000000	Kim et al (2019)	Tian et al. (2021)	34-9	SRR12791876	This study	DSM 2768 (HHO1)	SRR34702542, SRR34702543, SRR34702544, SRR34702545, SRR34702546, SRR34702556, SRR34702557
Hanseniaspora menglaensis	UCD88 (CBS 16921)	7	9.5	ASM4025685v1	Ryan et al. (2024)	This study	UCD88 (CBS 16921)	SRR34702547			
Wickerhamomyces anomalus	KG16 (primary haplotype)	9	13.7	JAHTLX01	Chun et al. (2021)	This study	CBS 5759	SRR34702548			
Cyberlindnera sargentensis	SHA 17.2	7	11.3	ASM2099542v1	Rueda-Mejia et al. (2022)	This study	SHA 17.2	SRR34702549			
Barnettozyma discipulorum	UCD2008 (PYCC 10016)	7	11.6	ASM4986350v1	Ryan et al. (2025)	This study	UCD2008 (PYCC 10016)	SRR34702555			
Barnettozyma californica	UCD09	7	12.0	PRJNA1289080	This study	This study	UCD09	SRR34702554			
Barnettozyma botsteinii	1118 (CBS 16679)	7	11.2	PRJNA1289080	This study	This study	1118 (CBS 16679)	SRR34702553			
Starmera quercuum	CBS 2283	7	11.7	PRJNA1289080	This study	This study	CBS 2283	SRR34702552			
Wickerhamomyces canadensis	CBS 1992	9	12.8	PRJNA1289080	This study	This study	CBS 1992	SRR34702550, SRR34702551			

References:

Chun BH, Han DM, Kim HM, Park D, Jeong DM, Kang HA, Jeon CO (2021) Metabolic Features of Ganjang (a Korean Traditional Soy Sauce) Fe mSystems 6:e0044121 pmid: 34342543

Kim JY, Kim J, Cha IT, Jung MY, Song HS, Kim YB, Lee C, Kang SY, Bae JW, Choi YE, Kim TW, Roh SW (2019) Community structures and geno J Microbiol 57:30-37 pmid: 30392155

Rueda-Mejia MP, Nägeli L, Lutz S, Ortiz-Merino RA, Frei D, Frey JE, Wolfe KH, Ahrens CH, Freimoser FM (2022) Genome sequence data of th Data in Brief 40:107799 pmid: 35071701

Ryan AP, Groenewald M, Smith MT, Holohan C, Boekhout T, Wolfe KH, Butler G (2024) Genome Analysis of a Newly Discovered Yeast Spec J Fungi (Basel) 10 pmid: 38535189

Ryan AP, Carvalho C, Zhao Y, Decuseara J, Osborne M, Heneghan PG, Byrne KP, Wolfe KH, Sampaio JP, Butler G (2025) Cyberlindnera hibe Int J Syst Evol Microbiol 75:006898 pmid: 40924470

Tian Z, Du Y, Yang F, Zhao J, Liu S, Zhang D, Long CA (2021) Chromosome Genome Sequencing and Comparative Transcriptome-Based Anal Front Microbiol 12:752529 pmid: 34858366
