# Supplementary material for: Centromeres in budding yeasts are conserved in chromosomal location but not in structure
Source: PLoS Genet. 2025 Dec 8;21(12):e1011814. doi: 10.1371/journal.pgen.1011814 (PMC12711049; doi:10.1371/journal.pgen.1011814)
Supplement: S2 Table — (PDF) [file pgen.1011814.s017.pdf]

Table S2. Genome assembly statistics for yeast newly sequenced in this study.

	B. botsteinii CBS 16679	*B. californica* UCD09	*S. quercuum* CBS 2283	*W. canadensis* CBS 1992
Number of nuclear contigs	7	7	7	9
Largest contig (bp)	2,330,928	2,856,532	3,102,089	2,801,313
N50 (bp)	1,705,254	1,748,282	1,840,606	1,608,033
L50	3	3	3	3
Total assembly length (bp)	11,193,175	12,038,359	11,673,475	12,760,144
