# Supplementary material for: Centromeres in budding yeasts are conserved in chromosomal location but not in structure
Source: PLoS Genet. 2025 Dec 8;21(12):e1011814. doi: 10.1371/journal.pgen.1011814 (PMC12711049; doi:10.1371/journal.pgen.1011814)
Supplement: S3 Table — Location of centromeres mapped by Hi-C in each species. For each scaffold, the number shown is the start position (in kb) of the 10-kb Hi-C peak window identified. (PDF) [file pgen.1011814.s018.pdf]

Table S3. Location of centromeres mapped by Hi-C in each species, and by ChIP-seq in *H. uvarum* .

**Species**	Scaffold number								
	**1**	**2**	**3**	**4**	**5**	**6**	**7**	**8**	**9**
Barnettozyma botsteinii (Hi-C)	1981	1635	1262	1147	276	112	84		
Barnettozyma californica (Hi-C)	1881	1955	1361	1387	1029	1014	247		
Barnettozyma disciplorum (Hi-C)	1782	414	630	733	1345	62	601		
Wickerhamomyces anomalus (Hi-C)	No CEN	331	No CEN	1121	1157	714	919	866	1296
Wickerhamomyces canadensis (Hi-C)	614	811	1095	853	563	No CEN	753	361	
Starmera quercuum (Hi-C)	1894	1659	1121	626	207	240	541		
Cyberlindnera sargentensis (Hi-C)	573	509	415	411	981	1047	110		
Hanseniaspora mengalensis (Hi-C)	1186	798	77	886	751	122	269		
Hanseniaspora uvarum (Hi-C)	1400	1121	721	No CEN	665	855	388	38	
Hanseniaspora uvarum (ChIP-seq peak location, start of 500 bp window)	1411.6	1121.9	723.7	No CEN	664.6	852.4	399.1	38.7	
Hanseniaspora uvarum (extent of the intergenic interval containing the ChIP-seq peak)	1410.5 - 1413.9	1121.0 - 1122.5	722.9 - 725.0	No CEN	664.6 - 665.4	851.3 - 856.1	398.8 - 400.5	38.4 - 39.6	

"No CEN" indicates that there is no centromere on these scaffolds, in a species that has more than 7 scaffolds, i.e. some chromosomes are fragmented in the assembly. For Hi-C data, the number shown for each scaffold is the start position (in kb) of the 10-kb Hi-C peak window identified, e.g. for *H. uvarum* scaffold 1 the Hi-C peak window starts at position 1400 kb and ends at 1410 kb. For the *H. uvarum* ChIP-seq data, the bottom two rows show the start position (in kb) of the ChIP-seq peak (a 500-bp window), and the coordinates of the intergenic region that contains this peak.
